# Recent Advances in Microneedle Platforms for Transdermal Drug Delivery Technologies

**DOI:** 10.3390/polym13152405

**Published:** 2021-07-22

**Authors:** Sipho Mdanda, Philemon Ubanako, Pierre P. D. Kondiah, Pradeep Kumar, Yahya E. Choonara

**Affiliations:** Wits Advanced Drug Delivery Platform Research Unit, Department of Pharmacy and Pharmacology, School of Therapeutic Sciences, Faculty of Health Sciences, University of the Witwatersrand, 7 York Road, Parktown, Johannesburg 2193, South Africa; sipho.mdandah7@gmail.com (S.M.); philemon.ubanako@wits.ac.za (P.U.); pierre.kondiah@wits.ac.za (P.P.D.K.); pradeep.kumar@wits.ac.za (P.K.)

**Keywords:** microneedle devices, transdermal penetration, drug delivery

## Abstract

In many clinical applications, the transdermal route is used as an alternative approach to avoid the significant limitations associated with oral drug delivery. There is a long history for drug delivery through the skin utilizing transdermal microneedle arrays. Microneedles are reported to be versatile and very efficient devices. This technique has spurred both industrial and scientific curiosity, due to its outstanding characteristics such as painless penetration, affordability, excellent medicinal efficiency, and relative protection. Microneedles possess outstanding properties for diverse biomedical uses such as the delivery of very large substances with ionic and hydrophilic physicochemical properties. Importantly, microneedles are applicable in numerous biomedical fields such as therapy, diagnosis, and vaccine administration. Microneedles are emerging tools that have shown profound potential for biomedical applications. Transdermal microneedle technologies are likely to become a preferred route of therapeutic substances administration in the future since they are effective, painless, and affordable. In this review, we summarize recent advances in microneedles for therapeutic applications. We explore their constituent materials and fabrication methods that improve the delivery of critical therapeutic substances through the skin. We further discuss the practicality of advanced microneedles used as drug delivery tools.

## 1. Introduction

To deliver drugs through the transdermal route, what is commonly used, are topical creams and hypodermic needles. Nevertheless, the low bioavailability of topical creams and the pain associated with hypodermic needles, present major challenges in transdermal drug delivery. Microneedle arrays have been explored to overcome the limitations imposed by these two classical methods of transdermal drug delivery [1]. Trypanophobia, more commonly known as needle phobia, is known as the fear of injections or hypodermic needles. It is estimated that approximately 10% of the adult population suffers from needle phobia, and it is far more common in adolescents between 5–16 years. In terms of immunology, hypodermic needle injections can raise the risk of infection and contamination in the event of accidental reuse and can cause hypersensitivity, swelling and bleeding at the site of administration [1]. In past decades, microneedle technologies have attracted much attention as injection tools that only minimally interfere with an individual’s biochemistry, during drug delivery applications, interstitial fluid sampling, and diagnostics. Microneedles enable the penetration of only a small area of the skin at a restricted depth, leading to minor inflammation of the dermal layers associated with discomfort and damage to the tissues [2]. In particular, microneedle innovations have gained preeminence in healthcare as they promise to eradicate needle phobia concerns. More importantly, microneedles counter several safety problems commonly related to the disposal of hypodermic needles. In this review, we explore the use of different types of microneedles for transdermal drug and vaccine delivery for a variety of disease conditions and highlight their advantages over other routes of therapeutic administration. We also discuss the diversity of materials used, including innovative methods applied to manufacture several types of microneedles for different therapeutic applications.

Microneedles are engineered to penetrate the epidermis and convey a drug directly into the subepidermal vasculature [3]. The explosion in drug discovery and production has been witnessed in recent decades. Innovation in drug delivery technologies have not only enabled new pharmaceutics to be successfully adopted but have also allowed new delivery methods to be developed for existing drugs [4]. The idea of microneedles was first recognized in 1976. However, they were impractical until the first application of microelectromechanical systems in 1998 and the American patent on the microneedle for transdermal delivery was issued synchronously granted [4,5,6].

The route of administration of a drug should be compatible with its physicochemical characteristics for enhanced therapeutic effectiveness and biocompatibility. Each form of administration has benefits and drawbacks, each requiring a particular design of delivery vehicles to overcome them [7]. The main routes of drug administration are oral, intravenous, intramuscular, and transdermal routes [7]. Oral administration is the most commonly used route for drug delivery since it is both convenient and economical. However, it is associated with restricted drug absorption caused by drug degradation from the gastrointestinal tract (GIT) microenvironment influenced by low pH and food. Moreover, the cytochrome P450 (CYP450) enzyme system’s hepatic first-pass effect greatly decreases the bioavailability of orally administered medicines [8,9]. The advantages of intravenous administration include a bypass of absorption barriers. Intravenous and intra-arterial drug administration normally provides, with minimal delay, high drug bioavailability and also the rate of administration can be easily managed to achieve a desirable constant plasma concentration [10]. On the other hand, intravenous and intra-arterial drug administration is the most hazardous routes of administration. This is because high concentrations of drugs may be delivered to organs as rapidly without control, thereby eliciting toxic effects which might be associated with severe pain [11]. An intramuscular injection is a procedure used to deliver a drug deep into muscles. This helps the drug to be easily absorbed into the bloodstream. However, injections are invasive, painful, uncomfortable, and risky for infection. Moreover, uneasiness during or after injection can discourage participation in occupational health programs if participation is optional [12]. Injection pain is due to the mechanical trauma caused by a needle puncture injury on nerve fibers [12]. Pain may also be induced by increased pressure from fluid deposition inside the tissues or sudden tissue distention from rapid fluid introduction [12].

Transdermal delivery of drugs is a painless strategy drug delivery which entails the application of a drug formulation to healthy skin. The off-putting factor is that hydrophilic drugs with higher molecular weights are limited by skin membrane barriers [13]. The arrival of microneedle-based transdermal delivery overcomes challenges such as patient compliance, discomfort, infection risk, restricted drug penetration concentration and long-term care compared to traditional delivery techniques [13]. Rationally, there are many inherent shortcomings in traditional drug delivery routes that, theoretically, can be solved by innovative drug delivery methodologies, such as microneedles technology [14]. Microneedles are designed to solely pierce the cornified layer and the viable epidermis devoid of reaching nerve endings and blood vessels; hence patients are not likely to feel massive pain during the procedure [15]. Emerging microneedle delivery systems have transformed the techniques of drug delivery and show great prospects in clinical applications. Table 1 compares various transdermal drug delivery systems.

## 2. Recent Research on Microneedles Arrays

Transdermal drug delivery provides a variety of benefits for patients, not only because it is non-invasive but also because it is convenient and presents other advantages, such as eliminating the first-pass metabolism and preventing gastrointestinal deterioration. Microneedle arrays have been shown to maximize the number of substances capable of transdermal delivery via breaching the stratum corneum and providing a channel for effective drug delivery into the epidermis. Microneedle arrays have been widely studied for the delivery of drugs and vaccines in recent decades, and patient diagnosis and management [17]. Scopolamine was the first drug delivered using a transdermal microneedle patch, that was approved in the United States in 1979 [18]. Thereafter, transdermal nicotine transdermal popularized the transdermal delivery technology for the general public. Herewith are the advantages of this technology: [17]

Hypodermic needles are most commonly used for transdermal drug delivery while topical creams deliver drugs to the skin surface with minimal penetration. However, hypodermic needles are not widely tolerated due to discomfort. The primary concern regarding transdermal patches is that certain drug molecules are unable to optimally penetrate the skin. The stratum corneum serves as a significant shield, allowing only some molecules, such as low molecular mass and lipophilic drugs to penetrate through it [16]. Microneedle arrays are known to be minimally invasive devices that penetrate through the stratum corneum membrane, thereby accessing the microcirculation of the skin and achieving systemic transmission through the transdermal pathways. Microneedles are reported with arrange of 50–900 μm in height, in different shapes and materials such as polymers, silicon and metals that are made by a technique known as microfabrication [19]. Their height is sufficient to reach the dermis, and minuscule enough to avoid puncturing dermal vasculature or stimulating dermal nerves [17]. Microneedles are subjected to the skin surface and painlessly pierce the epidermis, forming small aqueous pores from which drugs penetrate and spread to the microcirculation of the skin [17]. A microneedle tool is comprised of micron-sized needles, which are aligned on a small patch, incorporating the benefits of both the transdermal patch and the hypodermic needle [20]. Researchers have developed refined microneedle technology allowing hydrophilic and compounds with a high molecular weight to penetrate through the stratum corneum [20]. These characteristic aspects of this technology include quicker initiation of action (quicker administration), improved patient compliance, self-administration, better biodistribution and effectiveness [20]. To deliver drugs that are impermeable to the skin, microneedles create transient micropores across the stratum corneum. Micropore closure after microneedle-based drug administration is another critical aspect by which microneedles are evaluated because it influences the rate of drug diffusion to the skin microvasculature and interstitial fluid [21]. In a previous study, micro-projected pores were shown to close approximately 25% in the first 30 min in diameter and nearly entirely close after about 6 h [22]. According to Kalluri and Banga (2009), the duration for which the microchannels remain open is also an important factor which will affect the drug delivery. They further stated that in a hairless rat model after microporation, skin barrier function recovers within 2–3 h while pores close within 15 h in vivo [23]. Milewski et al. (2010) reported that the time frame in which restoration of barrier function occurs and the associated physiological processes are not well understood [24] and Bal et al. (2010) further confirmed that the pores do, in fact, close very rapidly after 15 min in most cases [25].

## 3. Classification and Fabrication

**Figure 1:**
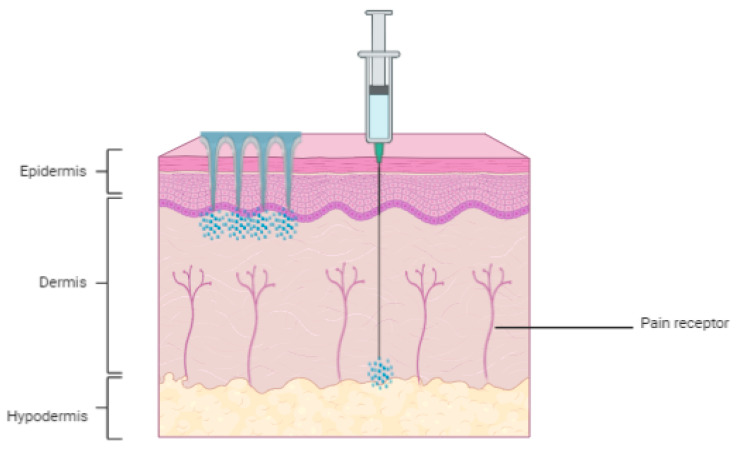
Drug dosing utilizing microneedle tools compared with a hypodermic needle.

The main objective of microneedles is to pierce the skin using micro-projections, without aching any nerves or causing injuries, thereby improving patient compliance and safety. Microneedles are also supported by patches, and they are designed to have a uniform, pressure-sensitive adhesive coating on one full side of the patch intended for interaction with the skin. Skin compatible adhesives are used to attach the microneedles securely on the skin surface to ensure they do not detach easily since the skin is flexible tissue and the microneedle substrates are rigid [26]. Microneedles can be categorized into different sorts based on numerous parameters which include drugs or biomolecules delivery methods, materials, and structural arrangement. Figure 1 illustrates a comparison of hypodermic needles and microneedles on how they penetrate the skin to deliver drugs.

Several microneedle designs are available in scale, tip shapes, length, diameter, and materials that contribute to the function of the drug delivery system. While microneedles can be used to target the skin as an almost painless route of delivery, not many prototypes display an acceptable degree of technical preparation for clinical applications. Numerous different prototypes of microneedles have been described in scientific literature, but the clinical assessment has so far been minimal. To commercialize any of these minimally invasive instruments on the market, an appropriate model must be created that helps clinical researchers to benchmark new microneedle procedures against traditional hypodermic needle injections [27]. Compared to other transdermal delivery systems, microneedles usually produce to a depth of 200 μm without entering the dermis, so there is no discomfort present [28]. Since 1990, great improvement has been made by the microelectronics industry, which is highly helpful for microneedle micro-manufacturing [6]. There are four types of microneedles, and they are classified as solid microneedles for the pretreatment of skin, dissolving and swellable microneedles without residual fragments, coated microneedles with water-soluble drug formulations, and hollow microneedles for liquid formulations. Table 2 shows a series of exemplary microneedle arrays and their functions [6]. 

## 4. Microneedles-Based Transdermal Drug Delivery Systems

### 4.1. Solid Microneedles

**Figure 2:**
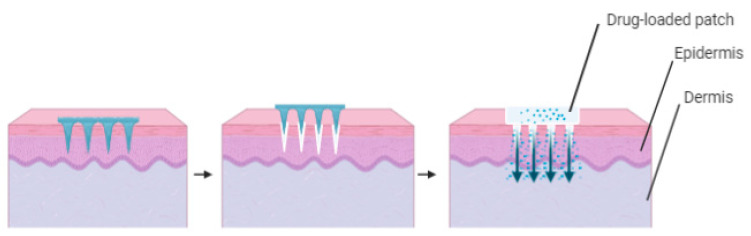
Solid microneedle device designed to create microchannel pores on the skin, then followed by transdermal patch application.

Solid microneedles puncture the stratum corneum and create microchannels pores. A drug formulation patch is then added to the skin for the drug to migrate eventually to the skin through the transient microchannels [36]. Solid microneedles are designed to deliver medications to the skin based on the “poke-and-patch” technique. In this technique, drugs in drug-loaded patches can be transported through diffusion or iontophoresis in case an electric field is applied. An alternative strategy is “coat and poke”, in which the microneedles are initially drug-coated and applied to the stratum corneum. In this approach, all the drug to be delivered is located on the surface of the needle as there is no drug reservoir on the surface of the skin. Another version of the second strategy is “dip and scrape”, where the microneedles are immersed into a drug or therapeutic substance solution and then scraped on the skin surface and leave behind the drug or therapeutic substance within the microchannels produced by the microneedles [37]. Figure 2 illustrates the functioning of solid microneedles.

In the literature, the first microneedle reported was impressed into a silicon wafer and formulated for in vitro intracellular delivery [37,38]. It was reported that to enhance molecular uptake and gene transfection, these microneedles were inserted into nematodes and cells. Subsequently, microneedles were then fabricated for transdermal drug delivery applications [38]. Currently, different kinds of solid microneedles have been developed. Narayanan and Raghavan (2017) developed solid silicon microneedles for optimized transdermal drug delivery with the following dimensions: an average height: 158 μm; a base width: 110.5 μm; an aspect ratio: 1.43; a tip angle: 19.4°; tip diameter: 0.40 μm [39,40]. In a seminal study, Martin et al. (2012) showed that sugar glass microneedles could be developed using sugar blend solutions in a vacuum at a low temperature. The microneedles were suitably structurally rigid to puncture human skin efficiently [41]. A previous study by Cha et al. (2014) reported that a microneedle array of polylactic acid (PLA: a biodegradable polymer) was produced by micro-molding polydimethylsiloxane (PDMS) [42]. Another study presented a microneedle porous titanium array (TPMA) made using a technique known as modified metal injection molding (MIM). TPMA was formed at 1250 °C for a period of 2 h. The biocompatibility of TPMA could be assured since only titanium and oxygen were found on the surface of TPMA. It was also emphasized that TPMA can penetrate through the skin surface without fracture or breakage [43].

The basic fundamental of microneedles involves minimal disruption to the skin layers, which produces micron-sized pores that aid the delivery of drugs directly to the epidermis or upper dermis. Drugs can then penetrate subepidermal blood vessels and enter the systemic circulation without being obstructed by the membrane barrier [16]. Microneedles range between 25 to 2000 μm in height, consisting of various materials and geometries. A variety of materials, including metals, silicone, glass, non-biodegradable, and biodegradable polymer materials, have been used to fabricate microneedles for therapeutic purposes [5]. Materials are the key determinants of the properties of microneedles such as strength, versatility, and permeability, and should be chosen wisely depending on the specific application. Microneedle devices can be manufactured by several types of materials such as ceramics, metals, silicon, glass and carbohydrates. These categories are summarized as in Table 3.

Several types of materials with desirable attributes, such as improved biocompatibility and high mechanical strength, have been explored for the manufacture of microneedles for transdermal therapeutic delivery. These include metals, silicon, glass, ceramics, and polymers such as carbohydrates. Metal microneedles display high mechanical strength, are easy to manufacture, and are produced using FDA-licensed medical devices made of relatively cheap metals, such as titanium, stainless steel, and nickel. The process of laser cutting, wet etching, metal electroplating and laser removal processes can be used to produce metal microneedles [26]. Due to its excellent characteristics, silicon is a very commonly used material for the manufacture of microneedles. Silicon has great mechanical strength and biocompatibility as the primary material utilized in micro-electromechanical systems (MEMS) [44]. The inertness of glass, its low cost and quick production makes glass-based microneedles good candidates for drug delivery applications [45]. Ceramic materials have been used for several decades in drug delivery. Porous ceramic microneedles permit the absorption and diffusion of drugs immediately after interposition in the interconnected pores. Its intrinsic porosity enables the microneedles to fill drugs without an additional processing step [44,46]. Micromolding can be used to fabricate microneedles composed of carbohydrates such as maltose, chitosan, trehalose, and starch. Carbohydrate-based microneedles are commonly manufactured using micromolding and drawing lithography [47]. Polymeric microneedles can be produced at room temperature by various methods, including tempering, micro-injection, or low-energy graph lithography. Polymeric microneedle arrays are particularly helpful for the administration of proteins, drugs, vaccines, and DNA. Polymer-based microneedles have swelling and dissolving properties with a polymer crosslinking network structure and hydrophilic characteristics [44,48,49]. Using the materials discussed above, microneedles can be fabricated to deliver drugs through various strategies.

### 4.2. Hollow Microneedles

**Figure 3:**
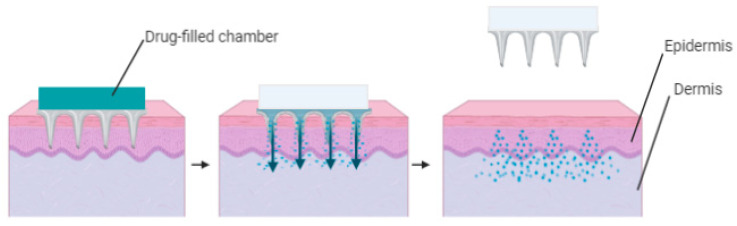
Illustration of poke and flow using hollow microneedles.

Hollow microneedles enable the movement of drugs from the patch reservoir to the microcirculation and can dose up to 200 μL volume [50]. In many ways, this tool mimics the function of a typical hypodermic syringe [50]. Hollow microneedles have holes at the tips and space which can be filled with a drug solution. The drug is immediately released into the epidermis or upper dermal layer when injected into the tissue (Figure 3). Molecules with a high molecular weight such as oligonucleotides, proteins and vaccines can also be delivered using hollow microneedles, [51]. The manufacture of hollow microneedles is considerably complicated and those with a high aspect ratio lack an internal support system similar to a solid needle, resulting in possible failure if incorrectly inserted. Careless handling of the patch assembly or unit, both during insertion and removal may result in stress which can lead to fracturing and failure of the needles [52]. Most microneedle fabrication approaches strive to decrease microneedle height and provide a more favorable safety margin [53].

Hollow microneedles provide a specific channel for the delivery of drugs to the skin or other tissues. The hollow microneedles, like hypodermic needles, aid in the adjustable, pressurized delivery of drugs in liquid form. [54]. In general, microneedles can become an extremely advanced medicine device and monitor for penetration of skin and perforation of the corneal barrier to the stratum corneum that allows for drug delivery into workable skin layers and the removal of body fluids. Despite the many years of research and the various types of MNs, only hollow MNs have reached the medical device market [55]. Over the past decades, advances have been made in the development of porous ceramic microneedles that can be used to deliver drugs at room temperature conditions [56]. 

### 4.3. Dissolvable and Swellable Microneedles

**Figure 4:**
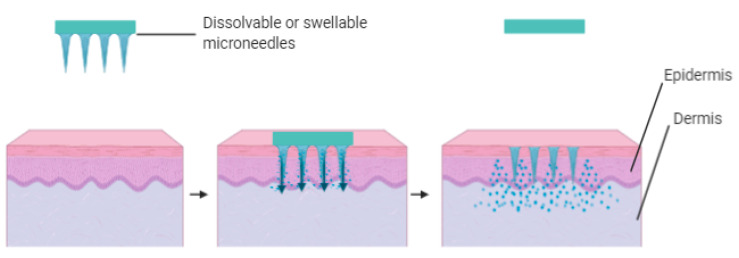
Illustration of poke and release using dissolving or swellable microneedles.

A recent technique to surface in microneedle fabrication is using swellable or dissolvable polymers. The drug to be administered is trapped within the needle at the fabrication stage. After penetrating the stratum corneum, the polymer forming the needle’s architecture dissolves and thus releases the entrapped drug (Figure 4). The benefit of dissolving microneedles inside the skin essentially reduces the risk of injuries due to needle-stick post-application [57].

Mikszta et al. (2009) stated that swellable microneedles (microneedles that swell after puncture of the skin) are more refined and seek to overcome the low-yield constraints of the dissolvable microneedles [58]. Swellable microneedle patches are based on the structure of a hydrogel, whose hydrophilic nature effectively extracts moisture from surrounding tissue, resulting in the widening of the microneedle core, forming pores into which the therapeutic substance can disperse [58]. The main benefit of this strategy is using the baseplate as a drug reservoir capable of carrying the underlying microcirculation via the swollen microneedle structure. Some recent advances in dissolving and swelling microneedle tools with a wide variety of drugs and polymers are illustrated in Table 4. They have a particularly innovative ability to remove interstitial fluid; it is a highly creative implementation of the swelling mechanism [58].

### 4.4. Coated Microneedles

**Figure 5:**
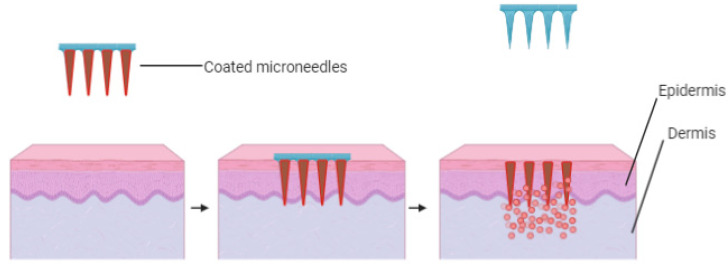
Illustration of “poke and release” using coated microneedles.

A coated microneedle array consists of sharp micrometer-sized needle shafts fixed to the base substrate and coated with a drug and water-soluble inactive excipients on their surfaces. Skin delivery of a variety of active materials, such as peptides, small molecules viruses and microparticles are constantly being evaluated for delivery using coated microneedles. The following factors are critical when considering the use of coated microneedles for drug delivery, quality of coatings, replicability of the coating procedure and the efficiency of drug delivery. In one study, the coating of an entire microneedles array plus the base substrate was made possible by immersing the microneedle patches in a coating solution [74]. Another study reported improved delivery efficiencies and reduced drug wastage by using specific coating methods to limit coatings to microneedle shafts only [75]. A coated microneedle is composed of a sharp, insoluble solid microneedle that is coated with active substances and water-soluble inactive excipients [76]. Utilization of hydrophobic coating material aids in the microneedle detachments when inserted in the skin and exposed to interstitial fluids [76]. The interaction with aqueous interstitial fluids dissolves the excipients in the microneedle coating, then the detachment process occurs on the microneedle surface. The rate of drug release within the skin relies on the interstitial solubility of the coating excipients. It is very important to remove the microneedles from the skin surface when the coating detachment process from the microneedle surface is complete [76]. Figure 5 illustrates the action of coated microneedles.

Single patches and arrays or multiple microneedle patches can be coated using a micron-scale dip-coating method [77]. The microneedle coatings have been developed by immersing the microneedle array in a coating and withdrawing it at an optimized speed. The study stated that the withdrawal speed of the microneedles from the coating solution was sustained manually at approximately 2 mm/s and 0.35 mm/s for film processing and pocket filling, respectively [78]. The coating uniformity was then evaluated using fluorescence or bright-field microscopy [26,78]. As mentioned before, although dip-coating is an old method, it is still important for coating microneedles due to its ease of use. However, surface tension (which is prevalent on the micron scale) and its effects, capillary and viscous forces, can affect the microneedle array substrate leading to an uncertain or diminished amount of drug [79]. In another study, the coating fluid was evenly applied to the surface of a 10 mm diameter roller to create a thin drug formulation film with a solution layer of approximately 200 μm. The microneedles were then horizontally connected to the cut-off device. The height of the tips of the microneedles was 50 μm lower than the top of the device. The roller was located at the top of the device and rotated at a linear speed of 0.3 cm/s. During the rotating process, the coating fluid was attached to the surface of the tips of the microneedles. The fabricated microneedles were vacuum dried and frozen [77].

Furthermore, the study reported a dip-coating process with 3D printed fixtures and microneedle plates. The preferred fixtures and microneedle plate were first drafted in Auto CAD software (Autodesk, Mill Valley, CA, USA) before being 3Dprinted. A polyformaldehyde plate was designed to separate the polylactic acid microneedles and the solution. To fabricate coated microneedles device, the parts were combined and then the microneedle shafts were dipped into the reservoir while being carefully monitored and controlled by a microscope connected to a computer. In the fabrication process, the portable holder went down at a pace of 10 mm/min, till it reached a coating solution from a reservoir. Microneedles were immersed then moved with a steady speed of 10 mm/min. Fixtured parts were dissembled, and the coated microneedles were observed by the microscope, vacuum dried and frozen [77].

## 5. Applications of Microneedles Technologies: Biomedical Applications

### 5.1. Microneedles Anticancer Agents

**Figure 6:**
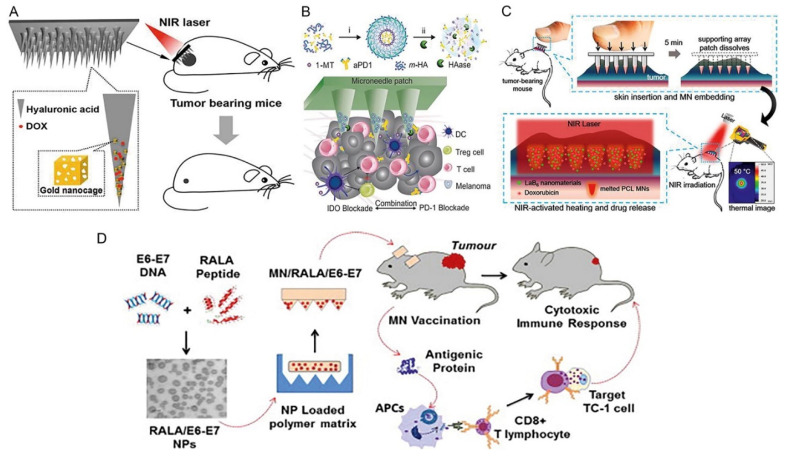
(**A**) Treating superficial tumors synergistically using chemotherapy and photothermal therapy. Dissolvable microneedles fabricated from hyaluronic acid (HA) were loaded with DOX. The drug-loaded microneedles were then incorporated with gold nanocages and exposed to near-infrared light, thereby combining chemotherapy and photothermal therapy. Adapted with permission from [83]. Copyright © 2021, American Chemical Society. (**B**) Microneedle-based melanoma immunotherapy. An anti-PD1 antibody was loaded onto an HA-based microneedle delivery system thereby combining PD1 and 1-MT to impede the enzyme, IDO, as an immunotherapeutic strategy for melanoma. Adapted with permission from (Wang et al., 2016a). Copyright © 2021, American Chemical Society. (**C**) Dissolvable PVA/PVP containing a polycaprolactone formulation was used to fabricate a light-sensitive microneedle patch. The polycaprolactone was composed of a chemotherapeutic drug, DOX, and photothermal nanoparticles which both acted in synergy for the treatment of dermal tumors. Upon exposure to near-infrared light, the microneedle patch melted at 50 °C and released DOX for local therapy. Reproduced with permission from [84]. Copyright © 2021, American Chemical Society. (**D**) The treatment of cervical cancer by applying a polymeric polyvinylpyrrolidone microneedle patch comprised primarily of the peptide RALA and DNA vaccine. Reproduced with permission from [85].

In recent years, cancer vaccines (immune- and gene-based therapies) have demonstrated encouraging anti-cancer effects and gained great interest from the scientific community [80]. Microneedles can penetrate the stratum corneum of the skin, typically above 200 µm deep, and release their drug contents upon interaction with interstitial fluid. This makes them important transdermal drug delivery vehicles in cancer therapeutics [81]. The insertion of hypodermic needles into the skin causes pain, irritation and generates hazardous wastes: side-effects which can almost be eliminated by microneedle-based drug delivery systems [16]. In addition, microneedles may contain the vaccine as a dried solid, which enhances its thermal stability and eases its administration to the target site [82]. Figure 6 shows some innovative microneedle-based anticancer therapeutic strategies.

### 5.2. Immune Therapies

Immune-based vaccines for cancer function by stimulating the host’s systemic immune reaction to eliminate the tumor tissue [86]. Immune-based methods typically investigate the delivery of vaccines to the skin, the body’s main immunological organ that is heavily populated with antigen-presenting cells (APCs), such as dendritic cells, macrophages, and Langerhans cells [87]. When activated, these APCs can activate the CD4+ and CD8+ T and B cells and hence stimulate a systemic antitumoral immune response [88,89]. Kim et al. (2019) fabricated a biodegradable microneedle patch that delivers a conjugate of hyaluronic acid (HA) and antigenic peptides for prophylactic cancer immunotherapy. HA was conjugated with cytotoxic T-cell epitope peptide (SIINFEKL), which was incorporated to a biodegradable HA microneedle patch to effectively deliver the antigen transdermally. The authors found that the HA-SIINFEKL conjugates loaded into microneedles were located near the site of administration of the microneedles, showing a long-term residence of above 24 h after delivery. In B16 melanoma model mice, tumor development was inhibited significantly through improved cytotoxic T-cell antigen-specific responses after only a single transdermal microneedle patch vaccination containing HA-SIINFEKL conjugates [90]. Considering recent developments in the treatment of melanoma using anti-PD-1 (aPD1) antibodies, there is still a need to enhance the effectiveness of this process (Wang et al., 2016a). The above authors reported a revolutionary biodegradable microneedle patch for the continuous and physiologically controllable distribution of aPD1. The microneedles consist of biocompatible HA-containing aPD1-encapsulating pH-sensitive dextran nanoparticles and glucose oxidase (GOx) that transforms blood glucose into gluconic acid. The low-pH environment generated facilitates the self-dissociation of nanoparticles and leads to the significant release of aPD1. Research by Wang et al. (2016a) showed that administering a single dose of the microneedle patch induced potent immune reactions in the B16F10 murine melanoma (skin cancer) model. In addition, this administration technique can be paired with an immunomodulator such as anti-CTLA-4 to improve the potency of an antitumor agent [91]. Considering the immense potential of DNA-based cancer vaccines, their successful delivery to antigen-presenting cells (APCs) to trigger the immune response is still a major obstacle (Irvine et al., 2015). While transfection by electroporation has enhanced efficiency, an ideal method for comfortable and painless vaccination is poorly understood. Irvine et al. (2015) reported a smart, nano-engineered DNA vaccine microneedle delivery system. The DNA vaccines were loaded onto microneedles coated with ultra-pH-responsive (polyethylene glycol-polyamino ester urethane ((OSM-(PEG-PAEU)) and a synthetic double-stranded RNA, poly (inosinic:cytidylic acid) (poly(I:C)), as an immunostimulatory agent. The study further stated that the engineered use of a vaccine and an adjuvant such as poly (I: C) in microneedles triggers immunity, which offers a promising vaccine technology that demonstrates enhanced effectiveness, conformity, and protection [88].

DNA can be easily transfected to APCs in the skin using dissolvable microneedles [85]. Nevertheless, this technique is characterized by the low transfection efficacy of pDNA and the small loading capability of microneedle systems. Distribution platforms incorporating microneedle systems and DNA delivery vectors show improved performance, but the problem of enhancing load capability persists. The research also stated that lyophilization was used to enhance the loading of RALA/pDNA nanoparticles in polyvinyl alcohol microneedles. Microneedle arrays preserve their structural and functional stability during short-term storage and can induce gene expression both in vivo and in vitro. Lastly, this innovative therapeutic formulation greatly slowed the development of existing tumors in a preclinical cervical cancer model and proved to be preferable to the conventional intramuscular injection [85]. Effective transmission of tumor antigens and immunostimulants to lymph nodes is essential for the maturation and subsequent activation of APCs, which further activates adaptive antitumor immunity [92]. Dissolving microneedle systems are considered desirable forms of transdermal immunization because of their excellent ability to administer vaccines via in a minimally invasive manner via the stratum corneum. Nevertheless, it is difficult to produce dissolving microneedles with poorly water-soluble vaccine components since they are typically formulated using aqueous-soluble polymers for speedy dissolution in intradermal fluids following administration.

The successful transmission of antigens to APCs, especially dendritic cells (DCs) and their consequent activation, is still a major difficulty in the production of efficient vaccines. Moreover, vaccine immunogenicity can be improved by using antigen-loaded microneedle arrays to target contiguous networks of DCs within the skin [93]. DCs in the skin could deliver antigen-loaded poly (d, l-lactide-*co*-glycolide) (PGLA) nanoparticles to skin-draining lymph nodes after in situ absorption, where they subsequently activated T cell expansion in an antigen-specific manner. It was demonstrated that microneedle vaccination of mice with antigen-loaded nanoparticles stimulated strong antigen-specific cellular immune responses [93]. Another study explored the in vitro microneedle-mediated transdermal delivery of human IgG as a model protein to deliver a monoclonal antibody [94]. Using methylene blue staining, microchannels created by the treatment of maltose microneedles with complete hairless rat skin thickness was visualized. In vitro penetration experiments were performed using freshly excreted, full-thickness hairless rat skin and different parameters such as needle length, number of needles and donor concentration effect were studied. Immunohistochemical (IHC) studies have traced the path of IgG transport across the skin. This study confirmed that the delivery of human IgG increased with the increase in microneedle arrays, microneedle concentration and length. In addition, maltose microneedles provided a means for the transdermal delivery of macromolecules [94].

### 5.3. Anticancer Therapeutic Drugs

Cancer remains a debilitating and deadly global disease against which multifaceted therapeutic strategies, including the use of microneedle arrays, have been developed. In a study by Uddin et al. (2020), a 3D-printed polymeric microneedle array was manufactured for improved delivery of cisplatin to A-431 melanoma tumors for cancer treatment. The microneedle arrays were produced using stereolithography (SLA), after which cisplatin formulation was coated on the microneedle surfaces. Optical accuracy tomography examination of the 3D printed microneedles displayed an impressive penetrating capability of 80% penetration depth. In addition, rapid rates of cisplatin release of 80–90% were shown in Franz’s cell diffusion studies in 1 h. Moreover, in vivo Balb/c nude mice were sufficiently permeated by cisplatin leading to enhanced antitumor activity and tumor regression. Using 3D-printed microneedle arrays establishes an efficient strategy by which anticancer drugs can be delivered transdermally, in vivo [95]. In another study, biocompatible and bioresponsive microneedles fabricated using gelatin methacryloyl (GelMA), loaded with the anticancer drug, doxorubicin (DOX), demonstrated sustained drug release and efficient transdermal therapeutic delivery [96]. DOX was loaded into GelMA microneedles using a single molding stage. The effectiveness of the DOX released from the GelMA microneedles was tested and the efficacy of the released melanoma cell line A375 drugs against cancer was demonstrated. Since GelMA is a flexible material for engineering tissue scaffolds and GelMA microneedles could reach the stratum corneum of mouse skin cadaver effectively, the GelMA microneedles could be used as a tool for the delivery of different therapeutics [97].

It is well known that even though cisplatin is a first-line chemotherapeutic drug, its clinical application is heavily limited by its systemic toxicity and adverse effects. A research study by Lan et al. (2018) showed that lipid-coated cisplatin nanoparticles (LCC-NPs) could be delivered transdermally using dissolvable microneedles for effective and safe anticancer therapy. Cisplatin showed a high rate of encapsulation of 80% into tumor-targeting, pH-responsive lipid nanoparticles. The high encapsulation rate greatly increased cisplatin solubility and improved its antitumor cytotoxic effect in vitro. The research further claimed that the LCC-NPs were trapped in dissolvable microneedles and released after they had been injected intradermally. This study showed that the cisplatin-nanoparticle microneedle system exhibits promising anticancer therapeutic properties by increasing cytotoxic anticancer effects and decreasing side effects [83]. Surgery and systemic therapy are common therapeutic interventions for superficial skin tumors (SSTs). However, surgery is comparatively invasive and systemic chemotherapy can induce several side effects [83]. However, topical treatment is faced with limited transdermal potential due to the barrier of the stratum corneum. It is therefore important to establish an efficient transdermal treatment strategy with minimal invasiveness for the treatment of SST. Dong et al. (2018) created gold nanocages (AuNC)-and DOX-charged hyaluronic acid dissolving microneedle arrays. In addition to improving the mechanical strength of the microneedles, the loaded AuNCs are also powerful agents for photothermal therapy against SST. The resulting MNs easily penetrate tissue, melt the skin, and expel lumps from the tumor location. The photothermal effect of AuNCs generated by near-infrared laser irradiation combined with the chemotherapeutic effect of DOX showed enhanced tumor cytotoxicity after four rounds of therapy on SST murine models. The drug/AuNC-loaded dissolving microneedles system, therefore, provides a promising forum for effective safe, minimally invasive combination SST treatment [83]. Frequent and multimodal therapies are required due to aggressiveness and the recurring existence of cancers. The risk of severe systemic toxicity and debilitating side effects is present in conventional cancer treatments. It is, therefore, important to create an alternative modality of anticancer therapy that is effective, shows minimal invasiveness, and displays low toxicity. Chen et al. (2016a) achieved synergistic anti-cancer cytotoxic effects on superficial tumors by designing a light-activating microneedle therapeutic device that can concurrently and repeatedly administer chemotherapy and photothermal therapy. This device consisted of polycaprolactone microneedles comprising a lanthanum hexaboride, a photosensitive nanomaterial, doxorubicin (DOX), and a protective collection patch of dissolvable poly(vinyl alcohol)/polyvinylpyrrolidone. The study proposed that the embedded microneedle array heats the target tissue equally to cause a massive thermal ablation region and then melts at 50 °C to unleash DOX in a wide area when exposed to near-infrared radiation, thus killing tumors. This light-induced heating and drug release activity can be specifically triggered for multiple cycles on- and off-demand [84].

### 5.4. Diabetes

Research using diabetic rats have shown that the transdermal ingestion of insulin from microneedles occurs within 1 h of exposure to the skin of rats in vivo after complete microneedle dissolution. In one study, which aimed at reducing the blood glucose levels in streptozotocin-induced diabetic rats, arrays of solid, insulin-infused microneedles were fabricated from a stainless-steel sheet and injected into their skin for the transdermal insulin delivery. As indicated by radioimmunoassay, the solid metal microneedles increased transdermal insulin distribution and lowered the levels of blood glucose in diabetic rats by 80% [98]. In another study, a dissolving microneedle patch was fabricated using gelatin and starch for effective transdermal insulin delivery [99]. Microneedles dissolve completely after they have been inserted into the skin for about 5 min, rapidly delivering their loaded contents into the skin. In one study, insulin-loaded microneedles were administered to diabetic rats to investigate the practicality of dissolvable microneedles in diabetes management [99]. The pharmacological availability and bioavailability of insulin were estimated to be 92%, showing that insulin maintains its pharmacological activity after being released from the dissolving microneedles [99]. Another research investigated skin perforation by commercially available microneedle rollers for the transdermal delivery of insulin to diabetic rats in vivo [100]. The research demonstrated that the rapid decrease in blood glucose levels in 1 h which was also directly associated with recovery, was caused by an increase in insulin permeability of the skin following the application of microneedle rollers. According to the study, microneedle rollers with a length of 500 μm or less are healthy and effective for in vivo transdermal insulin delivery [100].

Transdermal insulin delivery remains a critical problem because of low levels of therapeutically beneficial permeation. Research by Chen et al. (2009) reported that nanovesicles can be guided by iontophoresis to facilitate transdermal insulin delivery through skin microchannels created by microneedles. Insulin permeation concentrations from these nanovesicles powered by iontophoresis by microneedle-induced microchannel skin were several hundred times higher than those of passive diffusion [101]. In other research, the authors created new insulin-loaded microneedle arrays fabricated from hyaluronic acid (HA) for transdermal insulin delivery to rats. After dermal treatment, the insulin encapsulated HA microneedles with self-dissolving properties induce the rapid release of insulin. Pharmacokinetic and pharmacodynamic findings showed that the insulin delivered by HA microneedles was efficiently absorbed from the skin into the bloodstream. Moreover, the hypoglycemic effect induced by insulin-charged microneedles was almost identical to that of subcutaneous insulin injection. The data suggest that the insulin-charged microneedles made from HA represent an important alternative strategy of supplying insulin through the skin to the blood circulation without risking significant skin injury [102]. In an innovative research study, polymer-based microneedle patches were produced using a 3D printing process known as stereolithography for transdermal insulin administration [103]. The resin was photopolymerized to create pyramid and cone microneedle prototypes accompanied by inkjet printing of insulin formulations. Mannitol, trehalose, and xylitol were used as drug carriers to maintain the stability and integrity of insulin, while also promoting rapid release speeds. The insulin carriers retained their native form as shown by circular dichroism and Raman spectroscopy. Insulin was released easily, independently of the microneedle configuration, within 30 min. Overall, the data demonstrated that 3D printing is a successful technique for the manufacture of scalable and biocompatible microneedle patches [103]. In another study (Tong et al., 2018), a double-responsive insulin delivery system was developed to combine glucose- and hydrogen peroxide-responsive polymeric vesicles (PVs) with transdermal microneedle patches that showed excellent biocompatibility and painless administration. The drug-loaded polymeric vesicles effectively released insulin in response to hyperglycemia insulin and glucose oxidase (GOx) encapsulation. The rate of release of insulin reacted rapidly to increased glucose levels could be further enhanced by GOx, and induced hypoglycemia similar to that of subcutaneous injection or just insulin-charged microneedles. Hence, microneedle-mediated transdermal insulin delivery may be of considerable value for diabetic therapy [104].

In another study, calcium ion crosslinked alginate/maltose composite microneedles were developed by a template process for transdermal insulin administration in diabetic model rats. The alginate/maltose microneedles displayed a marked hypoglycemic effect with a higher relative bioavailability and relative pharmacological availability of 93.7 ± 4.7% and 94.1 ± 5.6%, respectively, when compared with the subcutaneous injection route [105]. Furthermore, in other research, polymer-based insulin-laden microneedle patches formulated with improved alginate and hyaluronate were produced and tested on diabetic mice to monitor their glucose levels in vivo using pharmacodynamic tests [106]. The insulin released from microneedle patches had a relative pharmacological availability and relative bioavailability of 90.5 ± 6.8 and 92.9 ± 7%, respectively. These findings indicate that the microneedles produced in this study have a promising use via transdermal delivery in diabetes care [106]. Even though proteins are effective biological therapies for treating different diseases, the transdermal delivery of protein therapeutics faces a major obstacle because of the low bioavailability and skin permeability. Research by Seong et al. (2017) introduced a new approach for the transdermal administration of proteins drugs using a double-layer, bullet-shaped microneedle array of water-swelling tips. Insulin was administered to the swelling tips utilizing a mild drop/dry process. In vivo, the sustained release of insulin from swellable microneedle patches led to a gradual decline in blood glucose levels [107].

In another study, an innovative microneedle drug delivery device which was fabricated by incorporating an insulin-loaded, hydrogen peroxide-responsive mesoporous silica nanoparticles displayed quick and pain-free delivery [108]. The hypoglycemic effect detected over time after transdermal injection into diabetic rats compared to subcutaneous injections revealed that the pre-prepared microneedles systems, H_2_O_2_, have promising applications in the treatment of diabetes [108]. A phase transition microneedle (PTM) patch is another innovative microneedle device that takes advantage of the unique virtue of polyvinyl alcohol (PVA) to form microcrystalline domains as cross-linking junctions. The PTM patch efficiently delivers insulin transdermally to the skin of pig models [109]. The study showed that insulin-charged PTM showed a transdermal bioavailability of more than 20% in the pharmacokinetic and efficacy trials using pig models The PTM patch could apply to several protein/peptide drugs which require regular dosing by providing painless management, freedom from refrigeration and limited concerns on safety [109].

### 5.5. Viral Disease

The administration of long-acting antiretroviral (ARV) medicines for preventing and treating human immunodeficiency virus (HIV) is one way to tackle its transmission. To create a discreet autonomous vehicle for delivering these ARV therapeutics, conformity with everyday oral regimes may be avoided. Developing options, such as intramuscular (IM) long-acting injections, involve daily access to health care and disposal facilities. This concept has therefore been established to test the use of the ARV candidate’s nanosuspension (LA) of rilpivirine dissolving microarray patches (MAPs) (RPV). MAPs are physically stable and can penetrate the skin, thereby delivering RPV intradermally. MAPs could enhance patient acceptability and conformity with HIV prevention and regimens. It could also battle incidents of needle-stick injury and blood-borne diseases transmission with wide-ranging benefits for those in the developed world in particular [110]. Acyclovir is commonly used to treat herpes labialis (cold sores), normally caused by Herpes simplex type 1 viruses (HSV-1). Topical acyclovir, however, is poorly effective as it is poorly permeable to the skin. In a research study, Pamornpathomkul et al. (2018) aimed to determine whether polymer microneedles can be dissolved to boost local acyclovir distribution. They developed water-soluble Gantrez S-97 mixtures with the dissolving microneedle arrays filled with acyclovir. After applying 0.089 N per needle force for 30 s, the microneedles penetrated neonatal porcine tissue, demonstrating adequate mechanical strength to stand compaction. Moreover, by using the microneedles, acyclovir accumulated at the basal epidermis, the target site of the herpes simplex virus, at up to 21.5 µg/cm^3^ in vitro, approximately five times greater than the 99% viral cytopathic effect inhibition (ID99) needed for HSV infections. The data demonstrated a promising approach to the successful locally delivery of acyclovir by acyclovir filled dissolving microneedle arrays [111]. In another study, a novel lamivudine (LAM) drug delivery system (NDDS) was designed to address the short-life disadvantages associated with LAM. Polymeric LAM-loaded nanoparticles were prepared to accomplish this aim and their transdermal delivery was investigated through passive and microneedle-mediated transport. Herein, nanoparticles were prepared using a double emulsion-solvent evaporation process using polylactic-*co*-glycolic acid (PLGA) and bovine serum albumin (BSA) as polymers and stabilizers. To prepare LAM-loaded nanoparticles, two separate LAM concentrations (10 and 20 mg/mL) were used (NP10 and NP20, respectively). Ramadan et al. (2016) recorded a steady-state NP20 stream values of 7.49 ± 1.46 μg·cm^−2^·hr^−1^ and 15.77 ± 1.5 μg·cm^−2^·hr^−1^ for pretreatment skin and the microneedle-related skin. They stated that the steady-state flow of LAM-loaded NP20 across the skin treated with microneedles was substantially higher than that of passive transport across untreated skin [112]. Patches of microneedles are becoming increasingly relevant as an alternate method for vaccine delivery. From 2007 through 2008, an approved seasonal influenza vaccine was developed using microneedles from TheraJect (VaxMat). Antigens mixed with trehalose and sodium carboxymethyl cellulose were embedded in the tips of the microneedles. The patches containing 15 μg per flu antigen strain were commonly characterized to affirm the stability of the antigen in microneedles after being produced. It became impossible to classify with the use of standard radial immuno-diffusion study through the presence of excipient and very low vaccine concentration on the microneedle plates. Kommareddy et al. further stated that new strategies such as the capture of antigens in the microneedle-patches, the enzyme-linked tests and enzyme digestion, accompanied by mass testing, were used. The in vivo mice immunogeneity of monovalent H1N1 at doses of 0.1 and 1 μg and trivalent vaccine at dose 1 μg were observed in mice after microneedle administration. The initial results from the mouse experiments are encouraging and demonstrate that the microneedle technology is feasible for the delivery of influenza vaccines [113].

### 5.6. Bacterial Disease

Microneedles are microscale projections used for the transdermal delivery of a vast variety of drugs, including antimicrobials. Dissolvable polymeric microneedles (DPMNs) are exciting transdermal drug delivery mechanisms with reduced invasiveness and increased patient compliance. The integration of a small amount of graphene oxide (GO) into biocompatible polymers for microneedle fabrication results in substantial new DPMN properties. These properties include significantly enhanced mechanical strength, increased moisture tolerance, anti-inflammatory and antibacterial properties, self-sterilization, and near-infrared light-activated controllers. These new properties increase their effectiveness and ease of use as transdermal products, enhance their ability to monitor drug release, extend the spectrum of polymers that can be used in the manufacture of DPMN, avoid microbial contamination during storage and transport, and minimize the risk of infection in clinical applications [114].

Another study revealed that a patch with the potential to prevent bacterial infections and facilitate tissue remolding is of great benefit for wound healing [115]. Chitosan, which is commonly used for wound healing, has many excellent qualities such as an inherent antibacterial property The authors utilized a chitosan microneedle biomass (CSMNA) to fabricate a patch with a smart, thermo-responsive drug delivery strategy to facilitate healing of wounds Chitosan, which is commonly used for wound healing, has many excellent qualities such as an inherent antibacterial property. The microstructure of microneedles also allows the efficient supply to this target area of loaded substances and prevents undue adhesion from the skin to the patch. Moreover, in the CSMNA micropores, the endothelial vascular growth factor (VEGF) is incorporated in a thermosensitive hydrogel. The smart release of pharmaceutical agents is thus controllable by a rising temperature triggered by inflammation at the wound site. It was shown that during wound closure, the CSMNA patch could facilitate angiogenesis, and tissue regeneration, making it potentially useful for clinical wound care [115]. Another research study assessed the use of hyaluronic acid (HA) and green tea extract (GT) antibacterial microneedles, to efficiently deliver green tea (GT) [116]. The researchers used a mold-based technology to manufacture a transdermal delivery system for antibacterial therapeutics using GT/HA microneedle patches with a maximum surface area of approximately 50 mm^2^. A decline of 95% of the growth of gram-negative (*S. typhimurium*) *Escherichia coli*, and *Salmonella typhimurium* and gram-positive bacteria (*S. Aureus*) and *Bacillus subtilis* [116].

Owing to its painless, non-invasive, and effective medical distribution methods, microneedles have become increasingly applied in different medical fields. However, their low adhesion and slow anti-microbial behavior continue to limit the functional applications in the various epidermal sites and habitats. In one study, hierarchical microneedles with multifunctional adhesives and antibacterial capacity were fabricated, based on Paenibacillen’s antibacterial techniques and adhesion mechanisms of mussel byssi and octopus tentacles [117]. The microneedles can suit the skin well, sustain a tight connection in dry, damp, or wet conditions, using polydopamine hydrogel as the base and a circle of suction-cup-built concentric chambers surround each microneedle; and conduct self-reparation after a break in doubles. As the hydrogel and polydopamine base load polymyxin, the microneedles have an excellent ability to withstand typical bacteria during storage and use, and high mechanical resistance. The micro-adhesives demonstrated excellent adherence and antibacterial function, not only when used in knuckles but also on osteoarthritis rat models. These findings demonstrate that bio-inspired microneedles break through the constraint of conventional methods and are perfect candidates for scalable transdermal drug systems [117]. As therapeutic agents have recently been reawakened, researchers have shown interest in the use of bacteriophages, especially for drug delivery, especially parenterally. However, phage injection has many drawbacks, such as management necessity and the risk of cross-contamination of healthcare providers. In this study, the transdermal administration of E. coli T4 bacteriophages in vitro and in vivo was based on new hollow microneedle poly(polycarbonate) (PC). Bacteriophage transmission was successful with microneedle in vitro via the full-skin thickness and dermatome skin. High levels of plaque formation per mL in the recipient compartment were established when transmitted over dermatome skin, and when delivered over a full-thickness skin, high concentrations were found in the receiver compartment. In in vivo experiments, the first microneedle-mediated phage administration resulted in 4.13 to 103 plaque-forming units/mL being observed in blood after 30 min. Clearance soon happened, with systemic circulation fully eliminated within 24 h with phages predicted to occur in the absence of infection. Ryan et al. (2012) showed here that microneedle-mediated transmission helps systemic phage absorption to succeed. Thus, bacteriophage treatments now will provide a systematic alternate route of delivery [118]. A major problem in the healing process is the presence of bacterial biofilms in wounds. Permana et al. (2020) proposed a hybrid approach to enhancing biofilm penetration and specifically the distribution of doxycycline (DOX) to the infection site, using bacteria-sensitive nanoparticles (NPs) made from poly(lactic-*co*-glycolic acid), poly(poly-caprolactone) and chitosan to fabricate dissolvable DOX microneedles. With the integration of these NPs in microneedles the dermatokinetic profiles of DOX were dramatically improved by higher retention times comparison with needle-free patches [119].

### 5.7. Ocular Microneedle Delivery

**Figure 7:**
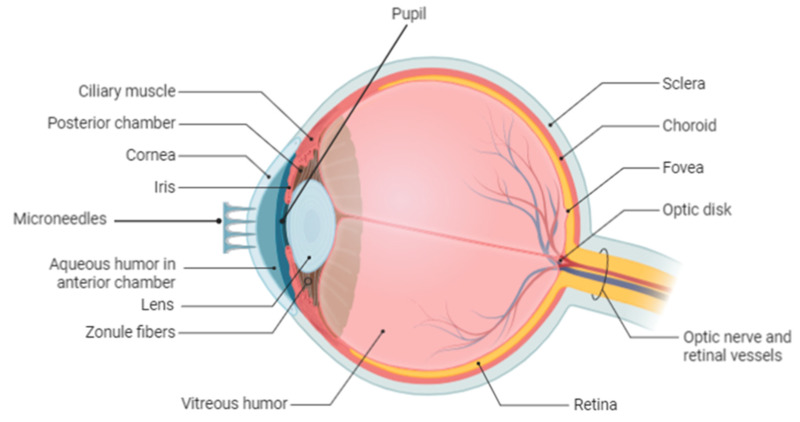
Human eye: anatomical features with the microneedles array.

Eye disorders and accidents are a major clinical concern worldwide. However, the safe and efficient delivery of eye medications is difficult due to the existence of eye barriers. Most eye diseases are treated with the use of eye drops or eye ointments, which have significant disadvantages such as frequent administration, reduced bioavailability, and the inability to cross various eye barriers. An alternative novel delivery system such as microneedles aims to provide treatments with promising health outcomes for diverse eye diseases. Developments in pharmaceutical technology have resulted in microneedles providing localized, effective, less invasive, and targeted delivery of drugs to the eye [120]. Figure 7 shows the cross-section of the eye and the region for microneedle application.

There is a critical unmet need to develop non-invasive, simple, and effective strategies for ocular drug delivery. Solid microneedles possess great potential in these aspects. Nevertheless, current microneedle devices have a limited drug-carrying capability owing to the broad spectrum of sizes that may be needed to deliver therapeutically appropriate doses. This may restrict their potential for clinical ocular delivery [118]. To overcome this limitation, titanium deep reactive ion etching (Ti DRIE) was used to generate microneedles with complex fenestrations or windows, which function as reservoirs for passive drug delivery. It was demonstrated that the developed microneedles devices showed sufficient stiffness for reliable insertion into ex-vivo rabbit corneas. In addition, it has been shown that these tools can increase the drug loading ability of microneedles by up to five times compared with solid microneedles of similar size, and also increased the drug release in the sub-surface of the rabbit cornea [121]. The dissolving microneedles used by Thakur et al. (2016) were found to boost the delivery of ocular macromolecules. Microneedles were produced using different molecular weights of polyvinylpyrrolidone (PVP) polymer. Conical PVP microneedle arrays of approximately 800 μm in height with a base diameter of 300 μm, comprising the drug model, were produced. On average, the microneedle drug content ranged from 0.96 to 9.91 μg. The study further revealed that the use of microneedles across corneal and scleral tissues in vitro studies demonstrated substantial increases in macromolecule permeation compared with aqueous solutions that were topically applied. As shown by confocal images, the macromolecules developed tissue depots which resulted in a persistent permeation [122]. Detachable microneedle technology is highly attractive for the treatment of eye disorders such as keratitis or glaucoma due to its reduced invasiveness and consistent drug delivery [123]. Shortening the administration time during microneedle insertion into target tissues, remains an important issue, although different methods have been attempted using mechanical and chemical separation mechanisms. The researchers fabricated a quickly detachable microneedle pen (RD-MNP) featuring a porous sacrificial dissolving coating for instant separation of the tip. Water-soluble polymers are used in the sacrificial layer: poly(vinyl alcohol) (PVA) and poly(vinyl pyrrolidone) (PVP). They showed that the pore size and distribution of the sacrificial layer were controlled and that the separation of an RD-MNP with the optimum pore structures is immediate when the MNP is dipped into a phosphate buffer solution. In addition, gelatin phantom and porcine eye sclera were used to study the insertion properties of the system. A microneedle tip is almost instantly removed and embedded into the porcine sclera with the optimized sacrificial layer with the aid of impact insertion [123]. Enhanced targeting of drug delivery to the posterior region of the eye is critical for the treatment of several post-segment eye conditions. Iontophoresis can be employed to transmit negatively charged nanoparticles to the posterior pole of the eye via the suprachoroidal space (SCS) [124]. The ex vivo injection of nanoparticles into the rabbit eye SCS without iontophoresis has been reported to contribute to the distribution of nanoparticles mainly located at the injection site near the limbus and <15% of the nanoparticles distributed to the most posterior SCS area (>9 mm from the limbus). However, the iontophoresis increased posterior targeting by >30% when the novel microneedle-based device was utilized. The study further reported that the treatment was well tolerated, with only moderate, intermittent tissue effects at the injection site. Using microneedle iontophoresis in the SCS promises to target the delivery of eye drugs within the eye, particularly to the posterior pole [124].

In another study, researchers used a technique in which an eye patch was fitted with detachable microneedle arrays containing micro reservoirs for regulated ocular drug delivery [125]. The researchers indicated that in a corneal neovascularization disease model, the transmission of anti-angiogenic monoclonal antibody (DC101) to such an eye patch induces a reduction of ~90% in the neovascular region. In comparison, the accelerated release of an anti-inflammatory agent (diclofenac) followed by a prolonged release of DC101 offers a synergistic clinical effect [125]. The application of an eye patch is simple and ensures good patient compliance due to its minimal invasiveness. Such an intraocular drug delivery strategy ensures successful home-based care for many eye disorders [125].

## 6. Conclusions and Future Perspectives 

The transdermal delivery of a wide variety of molecules has proven to be successful in a wide range of forms and designs. It is now possible to massively extend the spectrum of medications for successful transdermal delivery. This will greatly increase the importance of the transdermal delivery market which will become progressively relevant as the number of novel drugs continues to rise. Clinical trials on a small scale have highlighted the attractive attributes of microneedle-based devices, such as little discomfort, minimal invasiveness, mild inflammation if any and full regeneration of the skin within a few hours. In the monitoring of non-invasive therapeutic drugs/analytes, the possibilities for closure delivery systems may become significant. Microneedle technologies may also be used for further development. Focus group analyses define crucial areas for the advancement of the technology that must be explored by the Microneedles Ideology. This ensures that any patient will use reproducible microneedles and that successful insertion is verified. A substantial number of small and major industry players are currently engaged in clinical trials to commercializing their respective microneedle-based products. In future research, possible regulatory issues regarding the usage of microneedle devices will be discussed and the methods planned and developed to ensure low-cost, reliable means of mass production of microneedles. Overall, the future of the microneedles market looks to be very promising, with the rapid growth of the fundamental modern knowledge feed industry. It is hoped, in due course, that microneedles-based technical developments would contribute to improved disease detection, diagnosis and management, while at the same time enhancing the health-related quality of life for patients worldwide.

## Figures and Tables

**Table 1:** Comparative analysis and drug delivery applications of transdermal patches, hypodermic needles, and topical creams. Adapted from [16].

	Topical Cream	Transdermal Patch	Hypodermic Needle	Microneedle
**Description**	Creams and Ointments	Cohesive patch placed on the skin	Sharp tip with a small opening at the end	Microneedles fixed on the surface of a small patch
**Application**	Steady	Steady	Rapid	Rapid
**Pain**	Pain-free	Pain-free	Sore	Pain-free
**Bioavailability**	Sparse	Sparse	Good	Good
**Patient Compliance**	Non-compliant	Compliant	Non-compliant	Compliant
**Self-administration**	Yes	Yes	No	Yes
**Mechanism**	Permeation through the stratum corneum	Permeation through the stratum corneum	Drug impaled into the dermis	Drug bypassing the stratum corneum and directly into epidermis or dermis

**Table 2:** Microneedle materials employed for current technological application [6].

Type of Microneedle	Materials Used	Reference
Disposable	Carboxy-methyl-cellulose	[29]
Multi-round responsive	Alginate	[30]
Temperature-responsive	Vinyl pyrrolidone	[31]
Glucose-responsive	Hyaluronic acid	[32]
pH-responsive	Hyaluronic acid	[33]
Swelling-shrinking	Hydrogel	[34]
Water-soluble	Dextrin	[35]

**Table 3:** Materials for the fabrication of solid microneedles [39].

Materials	Advantages	Disadvantages	Application
Silicon	Biocompatible, hard, Mature fabrication techniques	Sharp waste Brittle	Solid, Coated, Hollow Microneedles
Glass	Chemically inert, Transparent and cheap	Cumbersome Fabrication, Brittle	Hollow Microneedles
Ceramic materials	Natural porous	Long fabrication Time, significantly brittle	Hollow, Dissolving Microneedles
Metals	Biocompatibility, High conductivity, have catalytic activity for some nanometals	High cost for noble metals, Allergic risk,	Solid, Coated, Hollow Microneedles
Polymers	Biodegradable (some) or Swellable, Easy fabrication	Low mechanical strength	Solid, Hollow, Coated, Dissolving, Swellable Microneedles
Carbohydrates	Biodegradable, Biocompatible	High processing Temperatures, low mechanical strength and hygroscopicity	Dissolving Microneedles

**Table 4:** Dissolvable/swellable microneedle devices. Adapted with permission from [59].

Drugs	Polymers	Types	Reference
Dihydroergotamine mesylate	Polyvinylpyrrolidone	Dissolving	[60]
Thymopentin	Polyvinylpyrrolidone	Dissolving	[61]
Exendin-4	Carboxymethylcellulose	Dissolving	[62]
Fluorescent Model	HA/PVA	Dissolving	[63]
Sumatriptan succinate	Polyvinylpyrrolidone	Dissolving	[64]
Adenosine	Hyaluronic acid	Dissolving	[65]
Vitamin K	Gantrez^®^ S-97 *	Dissolving	[66]
Lysozyme	Polyvinylpyrrolidone	Dissolving	[67]
Valproic acid	Carboxymethylcellulose	Dissolving	[68]
Besifloxacin	Polyvinylpyrrolidone	Dissolving	[69]
Caffeine/Theophylline	Hydrolyzed PEVE-MA and PEG	Swellable extraction of fluid	[70]
Glucose/Cholesterol	Methacrylated HA	Swellable extraction of fluid	[71]
FITC-dextran	Silk fibroin	Swellable	[72]
Curcumin	Gantrez^®^ S-97 PEVE-MA and Tween 85	Swellable	[73]

PEVE-MA: Poly(methyl vinyl ether-alt-maleic anhydride); PEG: poly(ethyleneglycol); HA: hyaluronic acid, * Gantr Gantrez^®^ S-97 is a copolymer of maleic acid and methyl vinyl ether.

## Data Availability

Not applicable.
